# N-Acetylcysteine in Combination with IGF-1 Enhances Neuroprotection against Proteasome Dysfunction-Induced Neurotoxicity in SH-SY5Y Cells

**DOI:** 10.1155/2016/6564212

**Published:** 2016-09-28

**Authors:** Benxu Cheng, Pinki Anand, Anxiu Kuang, Feroz Akhtar, Virginia L. Scofield

**Affiliations:** ^1^Regional Academic Health Center, School of Medicine, University of Texas Rio Grande Valley, Edinburg, TX, USA; ^2^Department of Biology, University of Texas Rio Grande Valley, Edinburg, TX, USA

## Abstract

Ubiquitin proteasome system (UPS) dysfunction has been implicated in the development of many neuronal disorders, including Parkinson's disease (PD). Previous studies focused on individual neuroprotective agents and their respective abilities to prevent neurotoxicity following a variety of toxic insults. However, the effects of the antioxidant N-acetylcysteine (NAC) on proteasome impairment-induced apoptosis have not been well characterized in human neuronal cells. The aim of this study was to determine whether cotreatment of NAC and insulin-like growth factor-1 (IGF-1) efficiently protected against proteasome inhibitor-induced cytotoxicity in SH-SY5Y cells. Our results demonstrate that the proteasome inhibitor, MG132, initiates poly(ADP-ribose) polymerase (PARP) cleavage, caspase 3 activation, and nuclear condensation and fragmentation. In addition, MG132 treatment leads to endoplasmic reticulum (ER) stress and autophagy-mediated cell death. All of these events can be attenuated without obvious reduction of MG132 induced protein ubiquitination by first treating the cells with NAC and IGF-1 separately or simultaneously prior to exposure to MG132. Moreover, our data demonstrated that the combination of the two proved to be significantly more effective for neuronal protection. Therefore, we conclude that the simultaneous use of growth/neurotrophic factors and a free radical scavenger may increase overall protection against UPS dysfunction-mediated cytotoxicity and neurodegeneration.

## 1. Introduction

In both sporadic and familial Parkinson's disease (PD), pathways leading to neuronal death are driven mainly by two key pathological events: aberrant protein homeostasis, including aggregation of proteins that normally would be disposed by the UPS system, and mitochondrial alterations secondary to proteinopathy-associated aggregation, which result from obligate mitochondrial Ca^++^ loading in response to this aggregation [1, 2]. A growing body of evidence implicates oxidative stress as an important intersection point between these two events [3]. Neurons are particularly vulnerable to proteinopathy (including abnormal aggregation of proteins) because of their long life span [4]. Death of dopaminergic neurons and accumulation of eosinophilic inclusions termed as Lewy bodies (LBs) in the substantia nigra pars compacta and other brain stem nuclei are the main pathological characteristics of PD [5]. During the development of PD, cellular defense mechanisms including antioxidants, the unfolded protein response (UPR), molecular chaperons, the ubiquitin-protease system (UPS), and autophagy are severely compromised [6]. Each of these defense systems offers therapeutic targets for intervention in the mechanism for their initiation and progression in the neurons.

In neurons, two particular protein clearance routes, the UPS and the autophagy pathways, are critical for the maintenance of cellular homeostasis, including ATP balance, amino-acid recycling, and protein quality control. Of these two mechanisms, UPS degradation of abnormal proteins is more important. The UPS process operates following “tagging” of abnormal proteins with ubiquitin, which in turn targets the tagged proteins to the proteasome for degradation [7]. However, treatment of cultured neurons with agents that cause proteasome inhibition results in compensatory autophagy, thereby preventing protein accumulation and cytotoxicity [7]. This suggests that clearance of proteins by the autophagy route correlates with their propensity to aggregate [8].

The LBs in substantia nigra neurons of PD patients are composed of a protein called *α*-synuclein, which is a substrate both for UPS degradation and for autophagy [7, 9]. Although neuronal autophagy appears primarily to be a protective process in the nervous system, it can also play a role in neuronal death because there is a limit to the number of autophagic vesicles (and their contained protein) that an individual cell can handle [8, 10].

The proteasome inhibitor MG132 (Carbobenzoxy-Leu-Leu-Leucinal), a membrane-permeable peptide aldehyde [11], effectively blocks the proteolytic activity of the proteosome complex. When the cell senses the buildup of aberrant proteins, its mitochondria begin to accumulate Ca^++^, resulting in cytochrome c release and mitochondrial dysfunction [12], which initiates PARP mediated cell death. Aberrant proteins also initiate ER stress, as indicated by the induction of CCAAT/enhancer-binding protein homologous protein (CHOP) expression [13], and the unfolded protein response, which leads to apoptosis.

In eukaryotic cells, the UPS is the main system that degrades misfolded proteins exporting from the ER. However, ER stress, as part of the UPR pathway, can also activate autophagy by conjugation of LC3 (microtubule associated protein 1 light chain 3) to membrane lipids [14].

In many neuronal cell types and in the Central Nervous System (CNS), proteasome inhibition causes mitochondrial dysfunction, reduces glutathione levels, and increases generation of free radicals [15]. This leads to the elevation of intracellular reactive oxygen species (ROS), resulting in oxidative stress. Increase in the levels of free radicals increases protein oxidation and further compromises proteasomal degradative capacity. By these activities, oxidative stress may fuel a vicious cycle that links mitochondrial dysfunction to proteasomal inhibition and autophagy, both of which lead to neurodegeneration [16].

Previous research has shown that antioxidant treatment of PD patients can mitigate neuronal damage by uncoupling mitochondrial damage from cellular oxidative stress in neurotoxicity and autophagy [16]. N-Acetylcysteine (NAC) is a membrane-permeable antioxidant and free radical scavenger that increases intracellular GSH by delivering cysteine to cells and acting as a precursor for glutathione synthesis. Its neuroprotective effects have been established in animal models and cell culture studies of non-PD neurodegenerative disease [16–19], and it has been successfully used for the treatment of psychiatric disorders [20]. In the present study, we assessed the individual and combined efficacy of two classes of agents, an antioxidant and a neurotrophic factor, for their ability to prevent cell death in cultured neuronal cells following UPS impairment with the proteasome inhibitor MG132. NAC was chosen as the antioxidative component and insulin-like growth factor-1 (IGF-1) was chosen as the neurotrophic component. The latter compound is a 70-amino-acid-long polypeptide with known neurotrophic, neurogenic, neuroprotective, and antiapoptotic effects, and it is essential for the development of the nervous system where it regulates cell growth, differentiation, and survival of neurons and their supporting cells [21, 22]. The primary mechanism by which IGF-1 elicits these efforts is the PI3/AKT pathway [23]. To the best of our knowledge, NAC and IGF-1 have not been tested for their ability, in combination, to protect proteasome-inhibited human dopaminergic cell lines.

## 2. Materials and Methods

### 2.1. Reagents

 N-Acetylcysteine, IGF-1, MG132, MTT [3-(4,5-dimethylthiazol-2-yl)-2,5-diphenyl tetrazolium bromide], and 3-methyladenine (3-MA) were purchased from Sigma-Aldrich (St. Louis, MO, USA) and Hoechst 33342 and 2′,7′-dichlorodihydrofluorescein diacetate (DCFDA) from Life Technologies (Eugene, OR, USA).

### 2.2. Cell Culture

Human SH-SY5Y cells and cell culture medium were purchased from the American Type Culture Collection (Manassas, VA, USA). The cells were cultured at 5% CO_2_ at 37°C in EMEM: Ham's F-12K medium (1 : 1) containing 10% fetal bovine serum (FBS), penicillin (100 units/mL), and streptomycin (100 *μ*g/mL). The cells were subcultured weekly in 60 mm or 100 mm cell culture dishes and used for experiments at 85–90% confluence. The culture medium was changed every 2-3 days. For Hoechst 33342 nucleic acid staining, cells were grown in 4-well chamber slides.

### 2.3. Inhibitor Treatments

The proteasome inhibitor MG132 was used to induce cytotoxicity in SH-SY5Y cultures. DMSO was used as a control vehicle, the final concentration of which was less than 0.1% in the cell culture medium. IGF-1 was suspended in sterile water, and NAC was dissolved in the culture medium. In neuroprotection experiments, SH-SY5Y cells were pretreated with IGF-1 (20 nM) and NAC (3 mM) alone, or with a combination of the two at the same concentration, for 18 h. The cells were then treated with MG132 (5 *μ*M) for 24 h, unless otherwise indicated. To avoid any reversible action, the inhibitors were maintained throughout the duration of the experiment.

### 2.4. Cell Viability Assays

The cells were incubated in 24-well dishes, and cell viability data was determined using MTT assays, measuring the conversion of MTT [3-(4,5-dimethylthiazol-2-yl)-2,5-diphenyl tetrazolium bromide] into formazan, as described in the previous study [24]. Thereafter, cells were treated with fresh media containing 0.25 mg/mL MTT for 2 h at 37°C. The medium was then removed and the purple formazan salt was solubilized with equal amounts of DMSO, and 100 *μ*L of each sample was transferred to 96-well plates. The spectrophotometric densities were determined at absorption of 490 nm. At least 6 replicates of each treatment were used.

### 2.5. Immunoblot Assay

Changes in the amounts of protein expression were measured by Western blot analysis. For Western blotting, the cells were lysed in ice-cold RIPA lysis buffer containing freshly added protease and phosphatase inhibitors. The lysates were clarified by centrifugation at 4°C for 20 min at 13,000 rpm, and their protein concentrations were measured with a BCA Protein Assay Kit (Pierce, Rockford, IL, USA). Equal amounts of the protein samples (25–30 *μ*g) were loaded and separated on 10% or 4–20% gradient polyacrylamide gels (Bio-Rad Laboratories, Hercules, CA, USA). The proteins were transferred to nitrocellulose membranes and blocked for either 1 h at room temperature or overnight at 4°C with Tris buffered saline containing 0.1% Tween 20 (TBST, pH 7.4) and 5% (w/v) skimmed dried milk.

Anti-PARP, anti-cleaved caspase 3, anti-CHOP and anti-LC3B antibodies were purchased from Cell Signaling (Beverly, MA, USA). Anti-ubiquitin antibody was obtained from Santa Cruz Biotechnology (Dallas, TX, USA). Anti *β*-actin was acquired from Sigma-Aldrich (St. Louis, MO, USA). The blotted membranes were incubated with primary antibodies for 1 h at room temperature or overnight at 4°C. They were then washed and incubated for 1 h with appropriate horseradish peroxidase-conjugated secondary antibodies. The protein bands were revealed using enhanced chemiluminescent (ECL) method, according to the manufacturer's instructions. Quantification of band densities was analyzed using one-way analysis of variance and Tukey's comparison test, using the Instat biostatistic program (GraphPad Software).

### 2.6. Cell Apoptotic Determination

An immunoblot assay was performed to assess PARP and caspase 3 cleavage for cells in the control and pretreated cultures. Nuclear morphological changes were assessed using Hoechst 33342 dye and visualized under a confocal microscope. For staining assays with the Hoechst 33342 nuclear dye, cells were cultured on glass-bottom dishes. After treatments, the cells were fixed with 3.7% paraformaldehyde for 20 min, washed with PBS three times, and stained with 1 *μ*g/mL solution for 30 min. Stained cells were mounted using Vectashield Mounting Medium for Fluorescence (Vector Laboratories, Burlingame, CA, USA) and imaged with an Olympus FV1000 confocal microscope utilizing Olympus FV10-ASW3.1 software.

### 2.7. Oxidative Stress Assay

ROS production was detected using a cell-permeant fluorescent dye, 2′,7′-dichlorodihydrofluorescein diacetate (DCFDA), as previously described [25]. After treatment, cells were loaded with DCFDA (10 *μ*M) and incubated for 30 min. The cells were then rinsed with PBS and fluorescence was measured immediately in a fluorescence microplate reader (SpectraMax, Molecular Devices). Wavelengths for excitation and emission were 485 and 520 nm, respectively.

### 2.8. Measurement of Reduced Glutathione

GSH levels were determined as previously described by Kamencic et al. 2000 [26]. Cells were cultured at 37°C in the presence or absence of the treatment reagents indicated above, washed with PBS, and incubated with monochlorobimane (MCB, 40 *μ*mol/L) in the dark for 30 min at 37°C. After two washes with PBS, fluorescence was measured by spectrofluorophotometer (SpectraMax, Molecular Devices), with excitation and emission wavelengths of 405 and 510 nm, respectively. Samples were assayed in triplicate.

### 2.9. Data Analysis

Our data was analyzed using one-way analysis of variance and Tukey comparison test, using GraphPad statistical software. Data is presented as the mean ± SEM of at least three independent experiments. In the figures, asterisks indicate the degree of significance for different treated cell cultures when compared to treatment controls or as specifically mentioned (^*∗*^ *p* ≤ 0.05–0.01, ^*∗∗*^ *p* ≤ 0.005, resp.).

## 3. Results

### 3.1. The Combination of IGF-1 and NAC Increases SH-SY5Y Cells Protection against MG132 Induced Toxicity

The cells were treated either with 3 mM NAC or 20 nM IGF-1 or with the two in combination for 18 hours, followed by exposure to MG132 (5 *μ*M) for 24 hours. The selection of dose and duration of exposure for IGF-1 and NAC were based on previously reported results [24] and preliminary dose effect data showing maximum protection, respectively.

**Figure 1:**
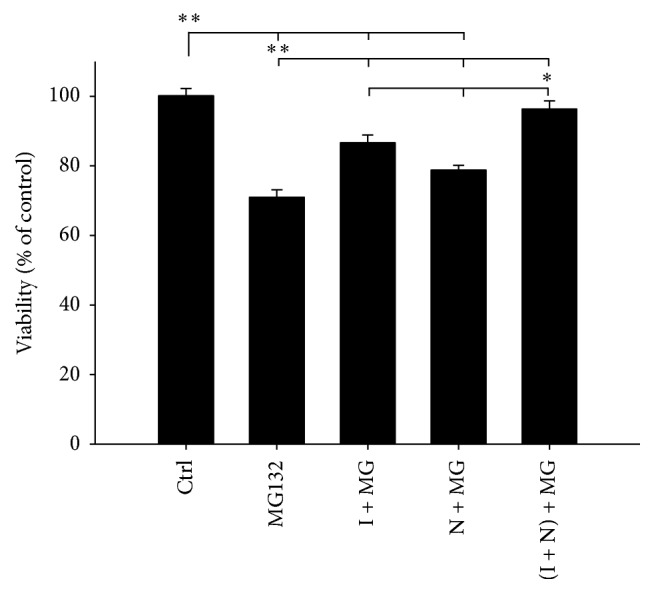
Pretreatment with IGF-1 and NAC increases cell viability after MG132 treatment. Cells were pretreated with vehicle (DMSO), IGF-1 (I), NAC (N), or both (I + N) for 18 h followed by the addition of MG132 (MG) (5 *μ*M) for 24 h. Cell viability was determined by MTT assay as described in the Materials and Methods. The results are expressed as percentage means ± SEM of control and are representative of three independent experiments with five replicates. Level of significance is denoted as ^*∗*^ *p* ≤ 0.05 and ^*∗∗*^ *p* ≤ 0.005.

As shown in Figure 1, MG132 treated cells showed significant reduction of cell viability, after 24-hour incubation, relative to untreated control cells (*p* < 0.005). In contrast, pretreatment of the cells with IGF-1 or NAC reduced MG132 induced cytotoxicity and promoted cell survival by 15.7%  ± 2.26% and 7.82%  ± 1.41%, respectively, compared to MG132 treated cells. Combined administration of NAC and IGF-1 produced the best protection of SH-SY5Y cells as evidenced by a 25.4% increase in cell viability (*p* < 0.005) (Figure 1).

### 3.2. The Combination of IGF-1 and NAC Attenuates MG132 Induced Apoptosis in SH-SY5Y Cells

**Figure 2 fig2:**
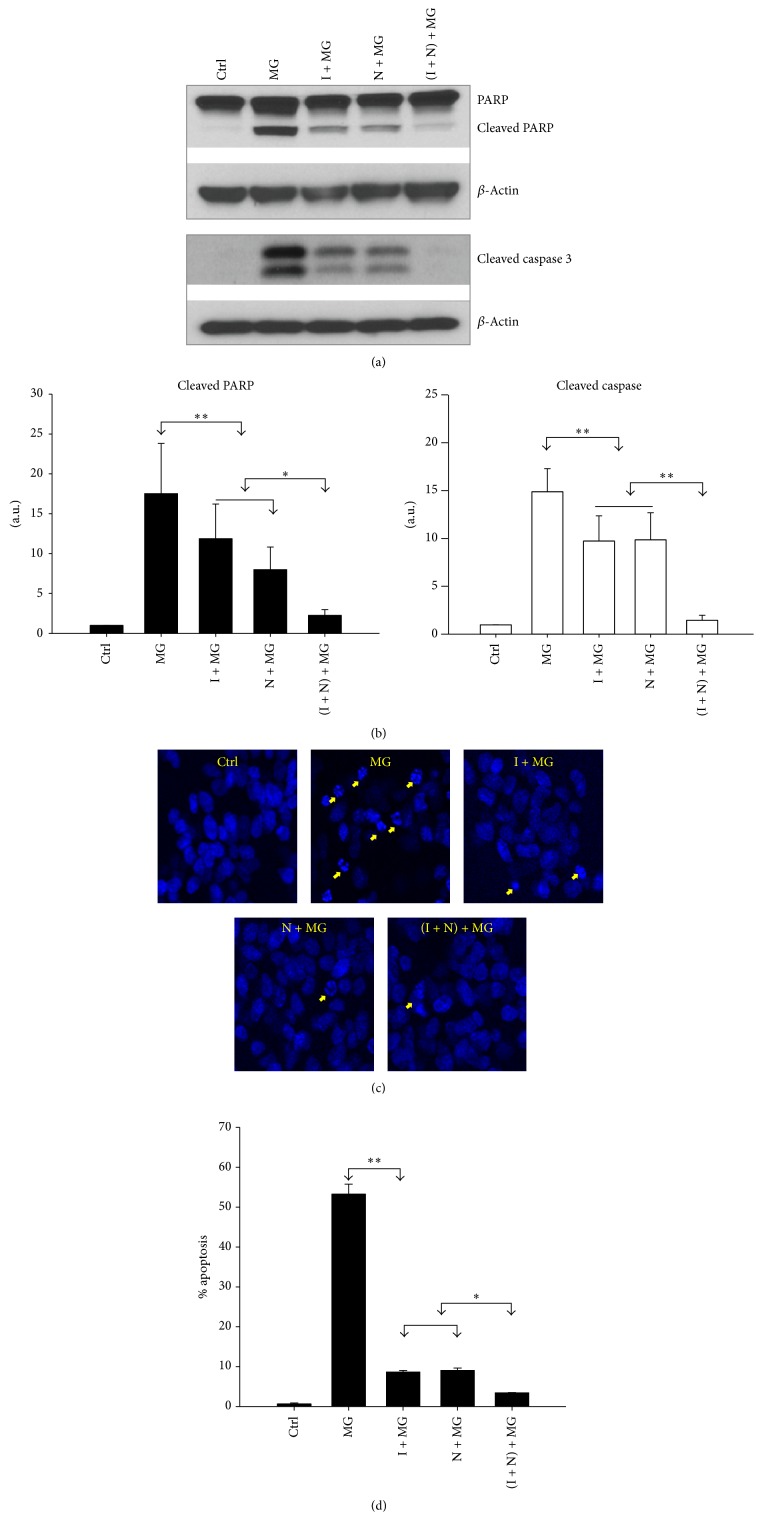
IGF-1 and NAC repress MG132 induced apoptosis. (a) Western blot analysis for two apoptotic markers: cleaved PARP and cleaved caspase 3. Results are representative of three independent experiments. (b) Densitometric analysis from three separate experiments. *β*-Actin served as the loading control. (c) IGF-1 and NAC prevent MG132 induced nuclear morphological changes. Cells were pretreated with IGF-1 (I), NAC (N), or both (I + N) for 18 h, followed by treatment with MG132 (MG) (10 *μ*M) for 24 h, as indicated. The treated cells were then stained with the Hoechst reagent. Nuclear morphological changes were imaged using a confocal fluorescence microscope (arrows denote apoptotic nuclei). The photomicrographs shown are representative of three repeated experiments. (d) Quantification of cells with abnormal nuclei. Values are mean ± SEM (*n* = 3) (^*∗*^ *p* ≤ 0.05, ^*∗∗*^ *p* ≤ 0.005).

Proteasome inhibition is known to be associated with induction of apoptosis [12]. To confirm and expand on the results from Figure 1, which suggested that NAC/IGF-1 are more effective together, Western blot analysis was used to detect two apoptotic markers, PARP cleavage and cleaved caspase 3 in treated and untreated cells. As shown in Figures 2(a) and 2(b), exposure of SH-SY5Y cells to MG132 for 24 hours resulted in significantly enhanced levels of both cleaved PARP and cleaved caspase 3 in comparison to control cells (*p* < 0.005). However, pretreatment of the cells with NAC or IGF-1 significantly reduced levels of both the markers (*p* < 0.05) in comparison to unprotected cells exposed to MG132. In agreement with Figure 1, the cells pretreated with both NAC and IGF-1 exerted complete protection against MG132 induced toxicity (*p* < 0.005) (Figures 2(a) and 2(b)).

In addition to the above apoptotic markers, we compared nuclear morphological changes between unprotected and protected cells. To visualize nuclear morphology, cells were stained with the DNA dye Hoechst 33342. Since lower doses of MG132, such as 5 *μ*M, do not cause evident chromatin condensation after 24 hours, a higher MG132 concentration of 10 *μ*M was employed. Cells incubated with MG132 alone exhibited extensive chromatin condensation and nuclear fragmentation, whereas relatively few of these structures were present in cells pretreated with NAC or IGF-1 alone. Furthermore, cotreatment with NAC and IGF-1 nearly completely prevented the appearance of apoptotic bodies in the cells (Figures 2(c) and 2(d)).

### 3.3. NAC and IGF-1 Alone or in Combination Attenuates MG132 Induced Reactive Oxygen Species (ROS) Generation and Depletion of Cellular GSH

**Figure 3 fig3:**
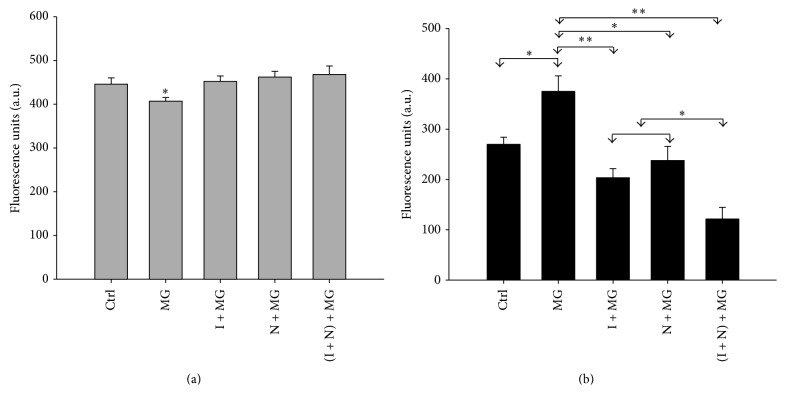
IGF-1 and NAC increase GSH production and reduce MG132 induced ROS generation in SH-SY5Y cells. Cell cultures were treated with NAC or IGF-1, separately or together, as described for Figure 1, followed by MG132 (5 *µ*M). (a) Glutathione assay results for the control and treated cells versus MG132 treated cells only. (b) Intracellular ROS levels. Level of significance is denoted as ^*∗*^ *p* ≤ 0.05 and ^*∗∗*^ *p* ≤ 0.005.

SH-SY5Y cells were untreated or treated with NAC or IGF-1 alone or together for 18 hours followed by addition of 5 *μ*M MG132 for an additional 24 hours as indicated in Figure 3. Thereafter the cells from all treatments were incubated with 10 *µ*M DCFDA or 40 *µ*M monochlorobimane (MCB) for 30 minutes, and their relative fluorescence was then measured. The results demonstrate that MG132 significantly reduced total intracellular GSH content (*p* < 0.05). However, MG132 induced depletion of GSH was attenuated by pretreatment with NAC or IGF-1, alone or in combination (Figure 3(a)). It was further noted that DCFDA intensity was elevated in cells treated with MG132 compared to the control. However, MG132 induced ROS production was significantly suppressed in cells pretreated with NAC (*p* < 0.05) or IGF-1 (*p* < 0.005) alone and was further reduced by the two together (*p* < 0.005) (Figure 3(a)).

### 3.4. Pretreatment with NAC and IGF-1 Does Not Reduce MG132 Induced Protein Ubiquitination, but It Inhibits Proteasome-Induced Autophagy

**Figure 4 fig4:**
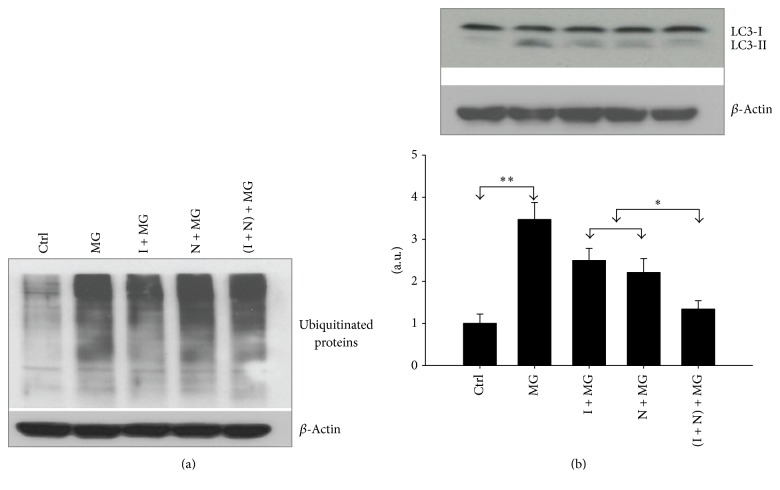
IGF-1 and NAC reduce MG132 induced autophagy without changing amounts of ubiquitinated proteins. After each treatment (indicated in the figure), total cell lysates were prepared for Western blot against ubiquitin and LC3B (an autophagy marker) antibodies. (a) Western blot for ubiquitinated protein. (b) Western blot analysis for LC3. *β*-Actin served as the loading control. Level of significance is denoted as ^*∗*^ *p* ≤ 0.05 and ^*∗∗*^ *p* ≤ 0.005.

Proteasomal inhibition causes accumulation of ubiquitinated proteins in large amounts [24, 27] irrespective of protection afforded by pretreatment with retinoic acid or IGF-1. Therefore, in the present study we sought to determine whether NAC, used alone or in combination with IGF-1, can reduce protein ubiquitination or affect autophagy, which is the default protein disposal pathway used by neurons whose proteasomes are dysfunctional [8].  Figure 4(a) confirms our previous finding from studies with other protective agents showing that proteasome inhibitor-treated cells contain significantly increased levels of ubiquitinated protein regardless of the presence or absence of NAC or IGF-1. To determine whether autophagy is deployed in NAC/IGF-1 protected cells, we tested challenged cells, protected challenged cells, and untreated cells for their levels of the autophagosomal membrane (lipid-bound) form of LC3. This marker is a mammalian homolog of the autophagy-related gene 8 in yeast [28] and is one of the most reliable markers by which to monitor autophagy [29]. Figures 4(a) and 4(b) show that MG132-treated cells contain increased levels of lipid-bound LC3 (*p* < 0.005). Though not significant, a partial reduction in LC3 level resulted in cells pretreated with NAC or IGF-1 alone. However, cotreatment with NAC and IGF-1 reversed MG132 induced expression of LC3 (*p* < 0.005). Furthermore, compared to treatment with either IGF-1 or NAC alone, cotreatment with NAC and IGF-1, LC3-II was further decreased (*p* < 0.05). These results identify autophagy as a central player in protein quality control, and in the subsequent cell death versus cell survival, of NAC/IGF-1 protected cells.

### 3.5. Cotreatment with NAC and IGF-1 Attenuates Endoplasmic Reticulum Stress

**Figure 5:**
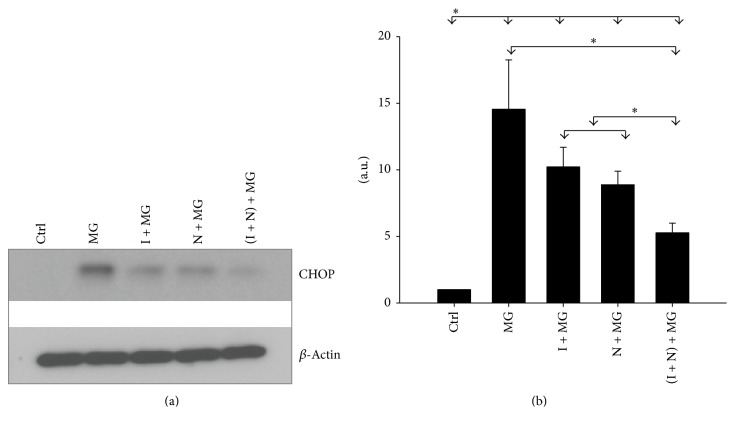
Pretreatment with IGF-1 and NAC represses MG132 induced ER stress. The cells were pretreated with IGF-1 and NAC, alone or together, for 18 h, followed by addition of MG132 (5 *µ*M) for 24 h. (a) Western blot analysis for CHOP (an ER stress marker). Data are representative of three separate experiments. (b) Densitometric analysis of CHOP from three independent experiments. Level of significance is denoted as ^*∗*^ *p* ≤ 0.05.

The involvement of endoplasmic reticulum (ER) stress in PD neuronal damage has been well-established [30, 31]. Suppression of proteasome activity induces the accumulation of CCAAT/enhancer-binding protein homologous protein (CHOP) in the ER [32]. To assess the degree to which ER stress participates in MG132 induced cell death of SH-SY5Y cells, we evaluated the effect of NAC and IGF-1 on expression of CHOP.  Figure 5(a) shows that exposure of the cells to MG132 results in the expression of CHOP. However, treatment of the cells with NAC, IGF-1, or both NAC and IGF-1 prior to MG132 exposure reduced its expression. In fact, cotreatment with both agents prior to MG132 exposure reduced the MG132 induced stress response to basal levels seen in the control. Densitometry analysis revealed CHOP levels significantly increased 14.5 ± 3 times (*p* < 0.05) in MG132 treated cells compared to the control, whereas the levels significantly decreased in cells pretreated with IGF-1, NAC, or both, respectively (*p* < 0.05) (Figure 5(b)).

### 3.6. Autophagy Inhibition Suppresses Apoptosis in SH-SY5Y Cells Treated with MG132

**Figure 6:**
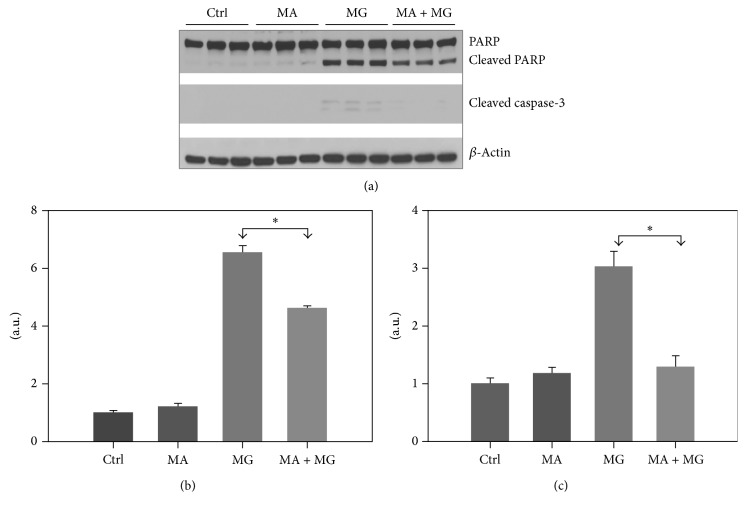
The autophagy inhibitor 3-methyladenine (3-MA) attenuates MG132 induced PARP and caspase 3 cleavage. The cells were pretreated with 3-MA (1 mM) for 1 h and then treated with MG132 for 24 h. (a) Western blot analysis for PARP, cleaved PARP, and cleaved caspase 3. Densitometric analysis for cleaved PARP (b) and cleaved caspase (c) from three independent experiments. Level of significance is denoted as ^*∗*^ *p* ≤ 0.01.

To investigate the contribution of autophagy to apoptosis, cell cultures were set up as described in Figures 1 [Fig fig2] [Fig fig3] [Fig fig4]–5, but in the presence or absence of the autophagy inhibitor 3-methyladenine (3-MA), which blocks autophagosome formation via phosphatidylinositol 3-kinase (PI3K). As shown in Figure 6, MG132 induced apoptosis is indicated by the expression of cleaved PARP (Figure 6(b)) and cleaved caspase 3 (Figure 6(c)); however, when cells were treated with the autophagy inhibitor 3-MA, both PARP and caspase 3 cleavage were dramatically reduced, suggesting that autophagy is partially involved in MG132 induced apoptotic cell death of SH-SY5Y cells.

## 4. Discussion

The efficacy of combination therapy depends on the use of drugs with different but complimentary mechanisms of action. Since PD pathology is driven by multiple factors, concomitant neuroprotective treatments are likely to be more effective than treatment with a single agent. Some well-established anomalies associated with PD are aberrant protein homeostasis, oxidative stress, excitotoxicity, and inflammatory processes, which induce apoptosis in the nigral dopaminergic neurons [33, 34]. Therefore, agents that could prevent these events could be neuroprotective, and their effect on disease progression, or even prevention, may be substantially enhanced when combination therapies are used.

The present study evaluates the neuroprotective potential of monotherapy and combination therapy of NAC and IGF-1 on proteasome dysfunction-induced neurotoxicity in human neuroblastoma SH-SY5Y cells by exploring the relationship between oxidative stress, ER stress, autophagy, and apoptotic cell death following proteasome inhibition. The neuroprotective effect of NAC is mainly due to its ROS scavenging potential and its maintenance of cellular GSH [16], whereas IGF-1 protects neurons against cell death via intracellular pathways involving PI3k/Akt-mediated phosphorylation FKHRL1 (a subclass of winged-helix family of transcription factors) [24, 35, 36] and inhibition of oxidative stress [37, 38]. A combination of both agents may broaden the spectrum of protection by acting on multiple mechanisms involving oxidative stress and multiple antiapoptotic signaling pathways.

We find that MG132 treatment substantially reduced the viability of SH-SY5Y cells. Evaluation of various parameters such as nuclear condensation and fragmentation and activation of caspases and PARP cleavage clearly indicated a pronounced proapoptotic effect stimulated by MG132. This cell death pathway involved ROS formation, GSH depletion, ER stress, and induction of autophagy. This study confirms, once again, that treatment of SH-SY5Y cells with MG132 can be a reliable model that replicates all the neuropathological abnormalities of PD, such as mitochondrial dysfunction, oxidative stress, and loss of proteasomal function [15].

While MG132 had a significant cytotoxic effect on SH-SY5Y cells as demonstrated by MTT assay, pretreatment with either NAC or IGF-1 protected cells from MG132 induced damage. Cotreatment with both agents resulted in a striking increase in protection. This effect may be the result of mitigating apoptosis, enhancing intracellular GSH, reducing ROS, and/or attenuating ER stress and autophagy. The antiapoptotic potential of NAC has been established both* in vitro* [12, 39] and* in vivo* [40]. Although the protective effect of NAC against MG132 induced cell death has been reported in various cell lines including neuronal cell lines, a similar* in vitro* dose of NAC did not attenuate the detrimental effect of MG132 in N27 cells [41]. This conflicting result may be attributed to the characteristic of the cell line used.

An important regulatory element in the protein ubiquitination pathway is the redox state of cellular GSH and its depletion may induce cell death [42]. MG132 induced cell death was preceded by pronounced depletion of GSH. Pretreatment of MG132 treated cells with either NAC, IGF-1, or both exerted similar effect of restoring the GSH levels. By acting as precursor to GSH and enhancing its biosynthesis NAC and IGF-1 maintain the reduced GSH content, respectively [43].

Pretreatment with NAC, IGF-1, or both significantly counteracted MG132 induced production of ROS. NAC acted as a direct scavenger and reduced more ROS in comparison to the individual treatments. Production of ROS is an intermediate step in MG132 induced apoptosis [44, 45] and thus protection of cells by NAC and IGF-1 pretreatment strongly highlights the key role of cellular redox status in the mechanism of neuroprotection by these two agents. Several lines of evidence also indicate the involvement of ROS and cellular redox status in the regulation of autophagy [46]. When we asked whether suppression of autophagy distinguishes protected, surviving MG132-treated cells from unprotected ones, we found that attenuation of autophagy is a defining feature of NAC/IGF-1-treated cells that survive MG132 challenge. While partial reduction of the autophagy marker LC3-II was detected in cells pretreated with either NAC or IGF-1, cotreatment of cells with both completely prevented MG132 induced autophagy, with the protective effects of either drug or both drugs being correlated with the degree to which the cells are protected.

Autophagy and ER stress are intimately related [47] and autophagy is accelerated in cells under ER stress [14]. In fact, impairment of autophagy by NAC or IGF-1 has been previously reported [16, 48]. Thus, the enhanced effect of NAC and IGF-1 demonstrates that combination treatment with these two agents has potential as a powerful therapeutic strategy against proteasome dysfunction-induced neurotoxicity. Furthermore, by demonstrating that autophagy plays a role in NAC and IGF-1 neural protection, our study implies that autophagy is a potential therapeutic target for PD treatment.

**Figure 7:**
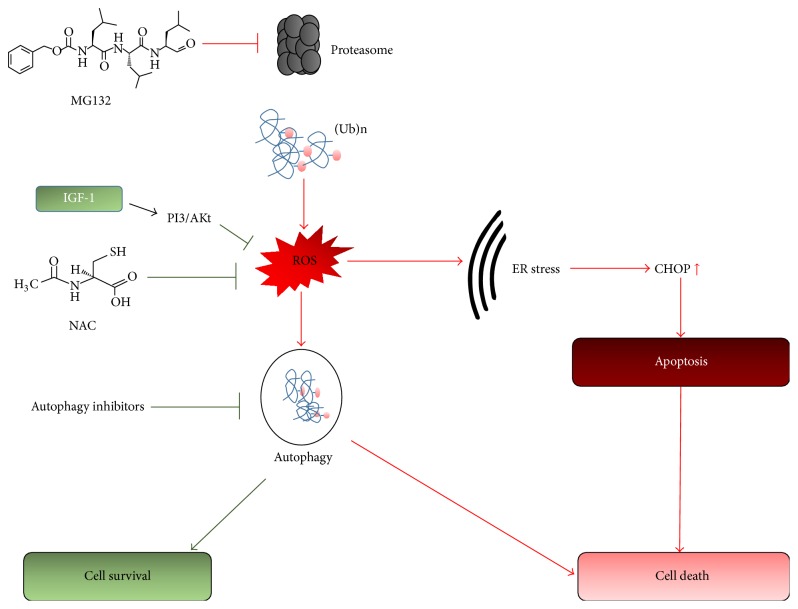
Schematic representation of molecular mechanisms of the proteasome inhibition induced cytotoxicity and its protection by NAC and IGF-1. Green lines represent cell survival pathways and red lines represent cell apoptotic and cell death pathways. Inhibition of proteasome by MG132 increases the aggregation of damaged and misfolded proteins. The accumulation of misfolded protein in the cytosol activates downstream ROS accumulation, ER stress, and cell death. Neuroprotective effect against MG132 of NAC and IGF-1 involves involvement of multiple protective pathways.

Despite considerable recent advances, it is still debated whether autophagy is either a cytoprotective response triggered by cells to survive cellular stress or a cytotoxic phenomenon associated with cell death [49]. Dysregulation of autophagy has been described in neurodegenerative diseases including PD [50, 51]. However, depending upon the cellular milieu, autophagy may promote cell survival or death [52, 53]. To assess the effect of autophagy in our system from a different angle, we measured MG132 induced cell death in the presence of an inhibitor of autophagy, 3-methyladenine (3-MA). Treatment with 3-MA partially blocked MG132 induced apoptosis (Figure 5), suggesting the involvement of autophagy in MG132-mediated neurotoxicity within SH-SY5Y cells. In summary, inhibition of proteasome by MG132 increases the aggregation of damaged and misfolded proteins. Acting as the primary cytotoxic stressor, the aggregated proteins then trigger downstream ROS accumulation, ER stress, and cell death via the caspase cascade and/or autophagy (Figure 7). The complete inhibition of apoptosis by the NAC and IGF-1, which only partially reduces LC3-II, underscores the involvement of multiple protective pathways involving ROS formation and GSH depletion and ER stress and autophagy.

## 5. Conclusions

Proteasome inhibition in SH-SY5Y cells triggers apoptotic cell death. A drug combination therapy comprised of NAC and IGF-1 completely alleviates cytotoxicity associated with proteasome dysfunction. The neuroprotective effect relies on modulation of multiple pathways associated with apoptosis, which include reducing ROS, GSH depletion, lowering ER stress, and preventing autophagy (Figure 7). Thus a combination therapy employing a neurotrophic factor (IGF-1) and a free radical scavenger (NAC) may provide a novel and promising therapeutic strategy for UPS dysfunction-mediated neurocell degeneration.
